# Blood Biomarkers for Assessing Headaches in Healthcare Workers after Wearing Biological Personal Protective Equipment in a COVID-19 Field Hospital

**DOI:** 10.3390/jpm11010027

**Published:** 2021-01-04

**Authors:** Francisco Martín-Rodríguez, Raúl López-Izquierdo, Raquel M. Portillo Rubiales, Laura N. Fadrique Millán, Virginia Carbajosa Rodríguez, Ancor Sanz-García, Guillermo Ortega Rabbione, Begoña Polonio-López, Miguel Ángel Castro Villamor, José L. Martín-Conty

**Affiliations:** 1 Advanced Clinical Simulation Centre, Advanced Life Support Unit, Emergency Medical Services, Faculty of Medicine, Universidad de Valladolid, 47005 Valladolid, Spain; fmartin@saludcastillayleon.es; 2 Emergency Department, Hospital Universitario Rio Hortega de Valladolid, Gerencia Regional de Salud de Castilla y León (SACYL), c/Dulzaina, 2, 47012 Valladolid, Spain; lfadriquemi@saludcastillayleon.es (L.N.F.M.); vcarbajosar@saludcastillayleon.es (V.C.R.); 3 C.S. de Tordesillas, Gerencia de Atención Primaria de Valladolid Oeste, Gerencia Regional de Salud de Castilla y León (SACYL), Crta. de Valladolid s/n, 47100 Tordesillas, Valladolid, Spain; rportillo@saludcastillayleon.es; 4 Data Analysis Unit, Health Research Institute, Hospital de la Princesa, Madrid (IIS-IP), C/Diego de León, 62, 28006 Madrid, Spain; ancor.sanz@gmail.com (A.S.-G.); agetro.ortega@gmail.com (G.O.R.); 5 Faculty of Health Sciences, Universidad de Castilla la Mancha, 45600 Talavera de la Reina, Spain; Begona.polonio@uclm.es (B.P.-L.); JoseLuis.MartinConty@uclm.es (J.L.M.-C.); 6 C.S. Delicias I Gerencia de Atención Primaria de Valladolid Oeste, Gerencia Regional de Salud de Castilla y León (SACYL), Pª Juan Carlos I, 18, 47013 Valladolid, Spain; mcastrovi@saludcastillayleon.es

**Keywords:** biomarker, COVID-19, headache, occupational health, personal protective equipment

## Abstract

The coronavirus disease 2019 (COVID-19) has led to a pandemic, which among other things, has highlighted biosafety as a key cornerstone in the management of disease transmission. The aim of this work was to analyze the role played by different blood biomarkers in predicting the appearance of headaches in healthcare workers wearing personal protective equipment (PPE) in a COVID-19 treatment unit. A prospective cohort study of 38 healthcare workers was performed during April 2020. Blood analysis, performed just before the start of a 4 hour shift, was carried out on all volunteers equipped with PPE. At the end of their shifts and after decontamination, they were asked if they had suffered from headache in order to obtain a binary outcome. The baseline creatinine value reflected a specific odds ratio of 241.36 (95% CI: 2.50–23,295.43; *p* = 0.019) and an area under the curve (AUC) value of 0.737 (95%CI: 0.57–0.90; *p* < 0.01). Blood creatinine is a good candidate for predicting the appearance of a de novo headache in healthcare workers after wearing PPE for four hours in a COVID-19 unit.

## 1. Introduction

The outbreak of coronavirus disease 2019 (COVID-19), caused by the SARS-CoV-2 virus at the end of December 2019, has led to a global public health emergency, with the subsequent declaration of a pandemic by the World Health Organization [1].

Since the start of this new infection, healthcare workers have been detected as the most affected group. On February 11, the Chinese Center for Disease Control and Prevention announced an infection rate among healthcare workers of 3.8% (1716 cases) [2]. Since the beginning of the pandemic, such data have highlighted the importance of wearing personal protective equipment (PPE) as one of the mainstays for the protection of professionals and to prevent the spread of the virus [3].

Working in an environment with a biohazard such as the current COVID-19 pandemic is complex, with high physical and psychological demands. Healthcare workers must be familiar with the scenario in which they are to work, but the healthcare system itself must also ensure the safety of its professionals and make sure they work with the utmost level of biosafety possible [4,5]. The correct use of PPE among healthcare workers has effectively resulted in the reduction of infections in other health emergencies [6], but it has also been seen that its use is not harmless for the professional, causing decreased field of vision; hearing alterations; increased feeling of warmth; reduced mobility; difficulty in breathing properly [7]; physiological consequences such as dizziness, nausea, vomiting, headaches, and hypoglycemic episodes [8]; and even psychological manifestations such as anxiety, stress, and bradypsychia [9]

COVID-19 treatment units are complex settings, which in many cases are unfamiliar to the workers and in which healthcare workers must perform precise and technical procedures using fine motor skills while wearing PPE [10,11]. If we add to these special working conditions the fact that a headache may occur, the working conditions become significantly more complicated, directly affecting the normal thinking process and influencing both decision-making and the outcomes of actions carried out, with resulting risks for the patient and the worker [12].

The use of prognosis biomarkers is a reality in clinical practice and a headache is no exception, with the roles of neuropeptides, cytokines, and adipokines having been studied [13]. These types of biomarker are very specific and difficult to process without appropriate equipment and specialist laboratory staff, so their rapid bedside use is not recommended. The development of small-scale and reliable point-of-care testing (POCT) has allowed for analyses in multiple clinical contexts [14]. In this sense, different biomarkers, for which POCT are already available, have shown their association with headache, for instance the evaluation of serum urea nitrogen and creatinine is necessary to rule out renal-failure-associated headache; hypercalcemia, hyponatremia, hypoglycemia, and dehydration have been also associated with headache [15,16,17].

The main objective of this study was to analyze the roles of different blood biomarkers (sodium, chlorine, calcium, potassium, creatinine, blood urea nitrogen, urea, lactate, and glucose) in order to predict the appearance of headaches when wearing personal protective equipment to deal with biohazards to healthcare workers in a COVID-19 treatment unit.

## 2. Materials and Methods

### 2.1. Study Design and Setting

A preliminary prospective study was carried out among cohorts of volunteer healthcare workers from a convalescence unit for patients with COVID-19 at a Valladolid field hospital (COVVA) between April 18 and 22. The study was conducted at the Advanced Simulation Center of the Valladolid University Faculty of Medicine (Valladolid, Spain).

The COVVA field hospital has 200 beds and was designed as a unit for patients with confirmed infection and initially good clinical progress. The facility was constructed in a 3800 m^2^ space with the highest biosafety standards. The field hospital has an electronic admission and medical history service, radiology and basic ultrasound, a laboratory, a pharmacy, an ambulance, and a hospitalization capacity of 200 patients.

The Research Ethics Committee of Rio Hortega University Hospital approved the study protocol (PI-075/20). All participants signed informed consent. This study was in accordance with Good Clinical Practice and the Declaration of Helsinki. The review protocol of this study was registered with International Clinical Trials Registry Platform ICTRP (doi.org/10.1186/ISRCTN18348009). This study is reported in line with the Strengthening the Reporting of Observational studies in Epidemiology (STROBE) statement [18].

### 2.2. Participants

Participants in the study were volunteers aged between 18 and 65 years old, were either nurses or medical assistants, and were all healthcare workers at the field hospital. Thirty-one patients were selected at random from an opportunity sample of 95 volunteers.

The exclusion criteria involved not signing the informed consent or having a headache or a temperature above 37.5 °C at the time of the study. Furthermore, volunteers with any of the following conditions were not eligible: body mass index greater than 40 kg/m^2^, major surgery in the last 30 days, taking anticoagulants or anticonvulsants, or systemic cutaneous or acute pulmonary diseases. Additionally, all professionals needed to follow the working rules for the COVID-19 zone of the field hospital, which included correct hydration (500 mL of water or isotonic drink) before entering the COVID-19 area.

### 2.3. Study Protocol and Measures

After signing the informed consent, the volunteers underwent blood analysis 15 min before starting their working day.

All samples were taken from the veins of the right antecubital fossa by the same registered nurse. The blood analysis was carried out using the epoc^®^ Blood Analysis System (Siemens Healthcare GmbH, Erlangen, Germany). The following biomarkers were collected sodium, chlorine, calcium, potassium, creatinine, blood urea nitrogen, urea, lactate, and glucose.

**Figure 1:**
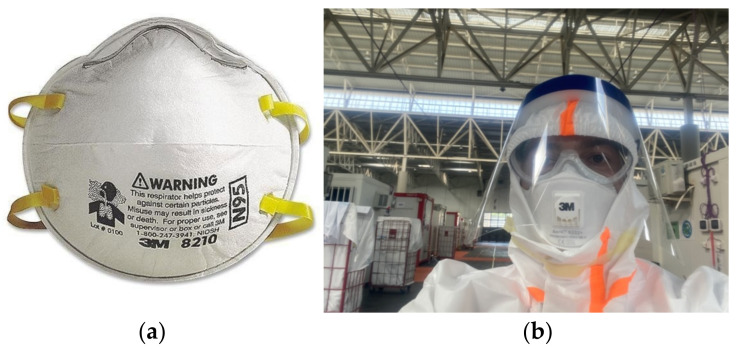
Face masks employed: (**a**) N95 face mask (source: Wikimedia Commons); (**b**) FFP2 face mask wore by a healthcare worker (FM-R), along with the personal protective equipment.

After the analysis, under the supervision of a biohazard specialist and in accordance with the standards of the European Center for Disease Prevention and Control, the professional was equipped with category-III PPE, type 4B/5B/6B [19]. The standard biological PPE was composed of a protective coverall, disposable gloves, non-powdered nitrile, panoramic glasses, and a transparent faceshield. In addition, the volunteers were randomly equipped with an Aura™ Face Mask 2 (filtering facepiece (FFP)) (3M, Saint Paul, MN, USA) or N95 face mask (3M, Saint Paul, MN, USA) (Figure 1). Table 1 shows the comparison between both masks.

Once equipped with the PPE, the professionals worked for a period of four hours before passing through a decontamination tunnel with a biohazard specialist then removing the PPE in a scheduled manner. Following decontamination and in a clean room, a registered nurse asked them if they had or have a headache. They were asked specifically about the nature of the headache, excluding any mechanical pain caused by the panoramic glasses, face shield, or face masks.

### 2.4. Outcome and Data Abstraction

The principal result variable was the presence of a headache after four hours of working with PPE.

Of all the cases within the range of study dates that met the inclusion criteria, the following were obtained: gender, age, professional category, time wearing the PPE, analytical data, and the presence of a headache.

All staff were aware of the objectives of the study and received specific information about the operation, cleaning, maintenance, and calibration of the analysis equipment. Each analysis was performed with a self-calibrating card with control of expiration dates, serial numbers, and batch numbers.

The data for all the participants were recorded electronically in a database created for this purpose. The analytical data were transferred via Bluetooth from the epoc^®^ Blood Analysis System to the principal investigator’s computer. To establish an accurate data link, the card’s serial number, age, sex, and time of analysis were linked to each test card.

### 2.5. Missing Data

Using logical, range, and consistency tests, a database was refined, which resulted in a total of 16 variables. A full variable-by-variable analysis of unknown data was then performed, leaving only full data sets for the analysis. The study variables did not present missing data. The case registration form was checked to eliminate ambiguous elements to guarantee the robustness of the data collection instrument.

### 2.6. Data Analyses

Normality tests were performed on all quantitative variables (Shapiro–Wilk and Lilliefors tests), showing that sodium, calcium, potassium, creatinine and lactate reflect normal distribution, with the other variables reflecting abnormal distribution; therefore, all the quantitative variables were described as the median and interquartile range (25th–75th percentile). The qualitative variables were described using absolute frequencies, with a confidence interval of 95% (CI95%).

The Mann–Whitney U test was used to compare quantitative variable measurements. The Chi-squared test was used for 2 × 2 contingency tables and for proportional contrast to stipulate the association or dependency relationship between qualitative variables. If necessary (percentage of cells with expected values less than five, greater than 20%), Fisher’s exact test was used.

The discrimination capacity of the different biomarkers was assessed using the area under the curve (AUC) of the receiver operational characteristics (ROC), calculating, in each case, the *p*-value of the hypothesis testing (H0:ABC = 0.5). The results for the ROC showed a CI of 95% after 300 resamples, as well as the best score offering joint greater sensitivity and specificity in each case. The positive predictive value, negative predictive value, positive likelihood ratio, and negative likelihood ratio were also calculated for these scores.

Also used was the analysis of variance (ANOVA) of the two factors in order to determine the possible interaction and principal effect of each factor appearing as significant in the univariate analysis.

The statistical analysis was conducted using IBM SPSS Statistics for Apple version 20.0. (IBM Corp, Armonk, NY, USA) and our own codes and base functions in R version 3.5.1 (http://www.R-project.org; the R Foundation for Statistical Computing, Vienna, Austria).

## 3. Results

**Figure 2:**
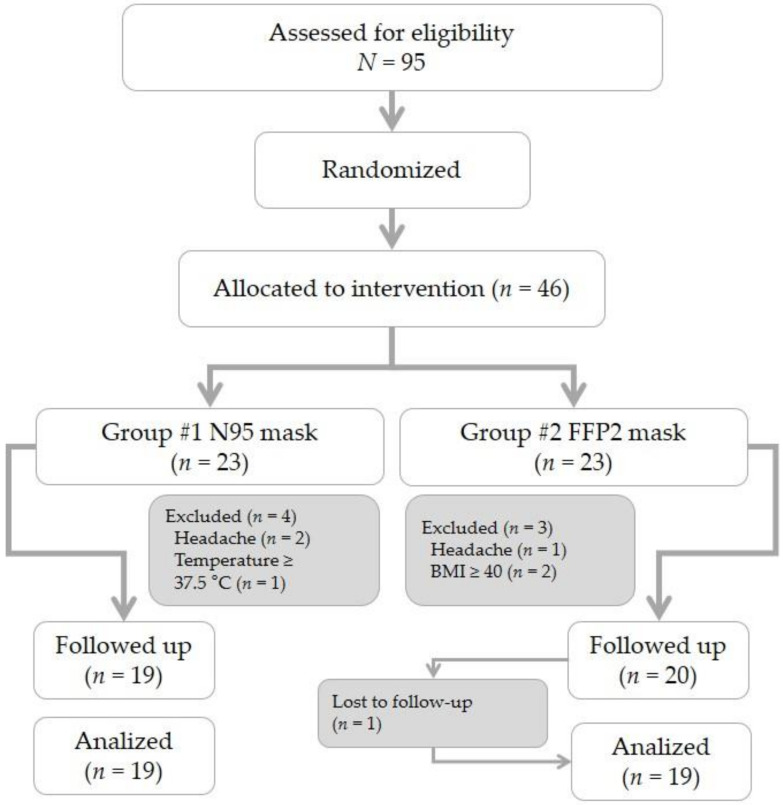
Flow chart of study population. FFP2: filtering facepiece.

Of a total of 95 volunteers that agreed to participate in the study, 46 participants were chosen at random and were then randomized based on the type of face mask to be worn during their work (N95 or FFP2). After exclusions, the final number of participants analyzed was 38 (Figure 2).

The median age was 29 years old (25th–75th percentile: 26–44 years). Here, 73.7% (28 cases) of participants were female. Nurses were the most common participants (15 workers, 39.5%), followed by physicians (12 workers, 31.6%) and medical assistants (11 workers, 28.9%), with a median of 4 years (25th–75th percentile: 3–8 years) of professional experience. The median time working with the PPE was 4 h 10 min (25th–75th percentile: 4 h 1 min–4 h 24 min). In total, 44.7% (17 cases) had a headache after wearing PPE for 4 h. Table 2 reflects the demographic characteristics and the analytical data for the two groups analyzed.

**Figure 3:**
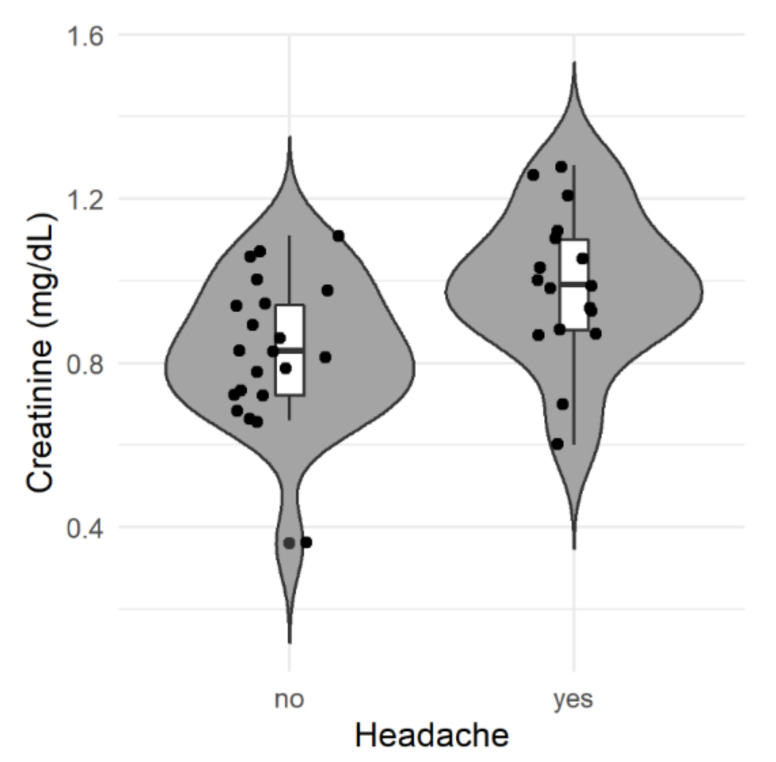
Distribution of creatinine levels according to the presence of a headache. The plot shows the distribution (gray shaded area), boxplot, and value of each patient’s (dots) creatinine levels from both groups (absence or presence of headache).

In the univariate analysis, only two significant variables were determined (creatinine and face mask type), as shown in Table 2. In terms of creatinine levels, it can be seen that the figures for this were higher in patients with headaches (Figure 3). Given that in the univariate analysis two variables showed a significant link with headaches, we proposed analysis of the possible interaction and principal effect for each of them. For this, a two-factor ANOVA (mask type and creatinine) was performed, which did not show significant interaction between the variables. A significant effect was only found for creatinine, so only that factor was considered for subsequent analyses (Table 3). The baseline creatinine value presented a specific odds ratio in the regression model of 241.36 (95% CI: 2.50–23,295.43), with a *p*-value of 0.019, demonstrating that the higher the level of creatinine, the greater the risk of headaches.

**Figure 4:**
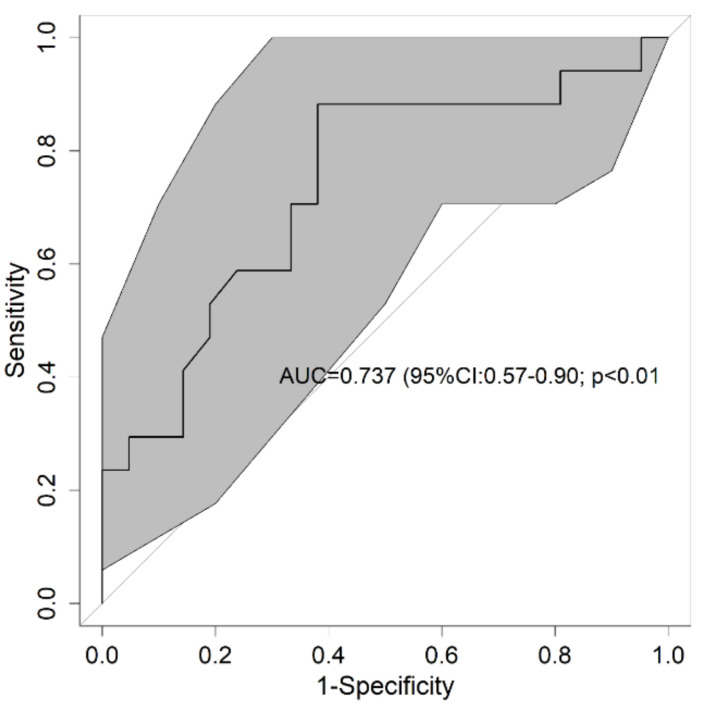
Receiver operational characteristics of creatinine for headaches. The bold line shows the value of the Receiver Operating Characteristic (ROC) curve and the gray shading is the result of 300 resamples. In the center of the graph is the area under the curve (AUC) and its 95% confidence interval and the *p*-value of the comparison against the null hypothesis (AUC = 0.5). ROC: receiver operational characteristics; AUC: area under the curve.

To determine the validity of creatinine in predicting headaches, the AUC was calculated and stood at 0.737 (95%CI: 0.57–0.90; *p* < 0.01) (Figure 4). In addition, the AUC was calculated to analyze the diagnostic capacity of creatinine based on the type of mask worn, with a value of 0.702 being obtained (95%CI: 0.46–0.94; *p* = 0.098) for the N95 face mask and 0.764 (95%CI: 0.49–1.00; *p* = 0.055) for the FFP2 face mask.

Table 4 shows the different characteristics derived from the ROC curve for creatinine and for the type of mask worn, with both cases being associated with the ability to predict headaches. In the case of creatinine, the cut-off point stands at 0.87 mg/dl, as can be seen in Figure 3, with two patients below this level in the group affected by headaches. In addition, creatinine has a high predictive value, as can be seen from the sensitivity and specificity reported in the table. However, the results observed for the two types of face masks are consistent with the results obtained and the lack of statistical significance in the ANOVA for the two factors (Table 3).

## 4. Discussion

Our results describe how a simple blood test providing creatinine values can predict with a high degree of certainty whether frontline healthcare workers at a field hospital during the current COVID-19 pandemic may develop a headache during their work wearing biological PPE, regardless of whether an N95 or FFP2 face mask is worn.

These results are in line with the findings of other investigators, in which headaches and neck pain have largely been associated with the wearing of various types of PPE in different working environments for healthcare workers [8,20,21].

However, it has been widely studied how wearing a face mask affects workers and may contribute to the onset of a headache [22,23,24]. In our cohort of subjects, a significant correlation was observed between having a headache and wearing an N95 mask compared with wearing an FFP2 during the analyzed work shift. In our cohort, over two-thirds of subjects wearing the N95 face mask developed a headache, while less than one-third of all subjects wearing the FFP2 mask developed a headache. Both types of face masks provide the same protection against biohazards [25,26]. The main difference between both devices is the fact that the FFP2 has an exhalation valve that can protect from the development of headaches [27,28].

Based on the blood test analysis, we can confirm that only the creatinine value prior to working in the COVID-19 zone has predictive value, with a high capacity for detecting the presence of headaches at the end of the work shift. Various studies have already explored this correlation between creatinine and the appearance of headaches with different results. Gozubatik-Celik et al. [29] measured creatinine before and after hemodialysis, but did not find differences between patients with and without headaches. Instead, Poyrazoğlu et al. [30] showed an increase of creatinine levels in an infant population diagnosed with migraine as compare to healthy subjects. Dehydration has been related to a headache increase [31] and also to a rise in creatinine levels [32]. However, this is not the case in our subjects, since blood samples were taken before their stay in the COVID-19 zone and adequate hydration was previously ensured at the start of the shift; moreover, the electrolytes results showed that dehydration could be ruled out. As with dehydration, changes in blood volume could affect the present results; this was solved by ensuring participants had a systolic blood pressure below 150 mmHg and above 80 mmHg before entering the COVID-19 zone. The measurement of creatinine using POCT is reliable, fast, and performed with a simple venous analysis or capillary blood sample [33,34]. In addition to our results, this may be helpful in anticipating and knowing which workers are more likely to develop a headache while wearing PPEs.

A headache is a factor that may have significant physical and psychological repercussions and which may directly affect healthcare professionals. In an ordinary working situation, the professional would communicate this situation, and if possible leave their workstation to treat the headache. In biohazard situations where biosafety is a priority, the problem is magnified—it is difficult to communicate the situation, decision-making is complicated or more complex, and decontamination and supervised removal of the PPE take priority, so knowing whether professionals suffer headaches during their work may be a very relevant piece of data.

Workers in biohazard situations must base their work on avoiding infections and the spread of pathogens. To achieve this objective, PPE is an essential tool, however it is not harmless to the workers, and physical or psychological effects may cause headaches, among others issues. Healthcare professionals must be in the best physical and psychological condition possible so as not to impair the decision-making process, meaning that workers predisposed to headaches must be considered by the healthcare system and that there must be clear strategies for PPE types and usage times.

Our study has several limitations. Firstly, the sample size was restricted due to the current pandemic, making access to larger cohorts difficult, and even more so in units devoted to caring for COVID-19 patients, where biosafety is paramount. For future studies and to generalize our results, multicenter studies are required with appropriate sample sizes. Secondly, there may have been a possible bias in participant selection. The sample was taken from among all workers at the Valladolid field hospital (COVVA) hospital. To minimize this bias, participants were selected with no stratification for gender, age, or professional category, and all volunteers were randomized to find out who would form part of the next phase of the study. There was no consideration of whether professionals had suffered from any type of chronic headache prior to the study. Finally, the analytical procedure may have been affected by inter-personal factors. To avoid this bias as much as possible, all staff involved in the study received a procedure manual and initial training on how to collect data and on the measurement and analytical instruments.

## 5. Conclusions

We present the use of blood creatinine markers for predicting the appearance of a de novo headache in healthcare workers after wearing PPE for four hours in a COVID-19 unit, regardless of whether an N95 or FFP2 face mask was worn.

A high incidence of de novo headaches was detected in healthcare workers after four hours of wearing PPE, which should be considered by healthcare systems in order to define usage times and the types of PPE to be worn, the profile of the professionals most suited to this work, and how to plan preventive strategies for them.

## Figures and Tables

**Table 1:** Comparison between the two face masks used.

	Mask Type	
	N95	FFP2
FDA-cleared	No	No
Exhalation valve	No	Yes
Model number (3M)	3M™ Particulate Respirator 8210, N95	3M™ Aura™ Particulate Respirator 9211+/37193(AAD)
Protects against airborne particles	Yes	Yes
95% filtration efficiency of aerosol particles	Yes	Yes
Latex	No	No
Price (Box of 10)	$12.99	$22.49

FFP2: filtering facepiece; FDA: Food & Drug Administration.

**Table 2:** Characteristics of the study population.

		Headache			
Characteristic ^1^	Total *n* = 36	No *n* = 21	Yes *n* = 17	Odds Ratio (95%CI)	*p*-Value
Age (years)	29 (26–44)	29 (26–42)	30 (27–45)	1.01 (0.95–1.07)	0.674 ^2^
Gender (female)	28 (73.7)	18 (64.3)	10 (35.7)	4.20 (0.88–19.94)	0.071 ^3^
Employment					
Physician	12 (31.6)	6 (28.6)	6 (35.3)		
Nurse	15 (39.5)	8 (38.1)	7 (41.2)	1.75 (0.32–9.29)	0.511 ^3^
M. assistant	11 (28.9)	7 (33.3)	4 (23.5)	1.53 (0.31–7.53)	0.600 ^3^
Mask type					
N95	19 (50.0)	7 (33.3)	12 (70.6)		
FFP2	19 (50.0)	14 (66.7)	5 (29.4)	4.80 (1.20–19.12)	0.026 ^3^
PPE time (hours)	4:10 (4:01–4:25)	4:10 (4:03–4:25)	4:05 (3:58–4:21)	1.00 (0.99–1.00)	0.640 ^2^
Blood test					
Na^+^ (mEq/L)	141 (140–143)	141 (140–142)	142 (140–143)	1.12 (0.77–1.62)	0.530 ^2^
K^+^ (mEq/L)	3.9 (3.6–4.0)	4.0 (3.8–4.0)	3.8 (3.5–4.0)	0.26 (0.02–2.63)	0.257 ^2^
Ca^++^ (mEq/L)	1.27 (1.23–1.29)	1.27 (1.25–1.29)	1.25 (1.23–1.29)	NA	0.495 ^2^
Cl^−^ (mEq/L)	104 (103–105)	104 (103–105)	104 (103–105)	1.08 (0.71–1.66)	0.701 ^2^
Urea (mg/dL)	5.1 (4.4–5.7)	5.1 (4.5–5.5)	5.0 (4.2–6.5)	1.38 (0.79–2.41)	0.252 ^2^
Crea (mg/dL)	0.91 (0.76–1.03)	0.83 (0.72–0.96)	0.99 (0.87–1.11)	241.36 (2.50–23,295.43)	0.019 ^2^
BUN (mg/dL)	12 (10–13)	12 (10–13)	13 (10–15)	1.11 (0.91–1.36)	0.287 ^2^
Glu (mg/dL)	96 (90–103)	96 (88–103)	96 (91–106)	1.00 (0.96–1.04)	0.936 ^2^
Lac (mmol/L)	1.13 (0.81–1.53)	1.13 (0.83–1.47)	1.14 (0.79–1.53)	1.07 (0.27–4.20)	0.914 ^2^

^1^ Values expressed as total number (fraction) and medians (25th percentile–75th percentile) as appropriate. ^2^ The *p*-values were calculated using the Mann–Whitney U-test. ^3^ The *p*-values were calculated using the Chi-square test. OR: odds ratio; CI: confidence interval; M: medical: Exp: experience; FFP: filtering facepiece; PPE: personal protection equipment; Na^+^: sodium; K^+^: potassium; Ca^++^: calcium; Cl^−^: chlorine; Crea: creatinine; BUN: blood urea nitrogen; Glu: glucose; Lac: lactate; NA: not applicable.

**Table 3:** Two-way analysis of variance (ANOVA) for type of mask and headache.

Characteristic	Degrees of Freedom	Sum Squares	Mean Squares	F Value	*p*-Value
Mask type	1	0.0950	0.09500	2.903	0.097
Headache	1	0.1606	0.16064	4.909	0.033
Mask*Headache	1	0.0013	0.00131	0.040	0.842
Residuals	34	1.1126	0.03272		

* Refers to interaction between factors.

**Table 4:** Measures of the predictive models for creatinine and headaches.

		Mask Type	
	Global	N95	FFP2
Headache prevalence	44.7	63.2	26.3
Creatinine cut-off point (mg/dL)	0.87	1.10	0.87
Area under the curve ^1^	0.737 (0.57–0.90)	0.702 (0.46–0.94)	0.764 (0.49–1.00)
*p*-value ^1^	<0.01	0.098	0.055
Sensitivity ^1^	88.2 (65.7–96.7)	41.7 (19.3–68.0)	100 (56.6–100)
Specificity ^1^	61.9 (40.9–79.2)	100 (64.6–100)	64.3 (38.8–83.7)
Positive predictive value ^1^	65.2 (44.9–81.2)	100 (56.6–100)	50.0 (23.7–76.3)
Negative predictive value ^1^	86.7 (62.1–96.3)	50.0 (26.8–73.2)	100 (70.1–100)
Likelihood ratio (+) ^1^	2.32 (1.31–4.10)	0	2.80 (1.39–5.65)
Likelihood ratio (-) ^1^	0.19 (0.05–0.73)	0.58 (0.36–0.94)	0
Diagnostic accuracy ^1^	73.7 (58.0–86.0)	63.2 (41.0–80.9)	73.7 (51.2–88.2)
Pretest probability	44.7	63.2	26.3
Youden’s test	0.5	0.4	0.6

^1^ Bracketed numbers indicate 95% confidence interval. FFP: filtering facepiece.

## Data Availability

The data presented in this study are available on request from the corresponding author. The data are not publicly available due to involve confidential clinical data of workers.
